# Development, Evolution, and Outcomes of More Anatomical Reverse Shoulder Arthroplasty

**DOI:** 10.3390/jcm13216513

**Published:** 2024-10-30

**Authors:** Pablo Sanchez-Urgelles, Logan Kolakowski, Jay M. Levin, Mark A. Frankle

**Affiliations:** 1 Foundation for Orthopaedic Research and Education, Tampa, FL 33607, USA; sanchezurgelles.pablo@gmail.com; 2 Department of Orthopaedic Surgery, University of Maryland, Baltimore, MD 21201, USA; 3 Rothman Orthopaedics, Philadelphia, PA 19107, USA; 4 Florida Orthopaedic Institute, Tampa, FL 33637, USA

**Keywords:** reverse total shoulder arthroplasty, cuff tear arthropathy, osteoarthritis, long-term outcome, implant design, biomechanics

## Abstract

Reverse shoulder arthroplasty (RSA) has become a widely used procedure since its introduction in the 1980s, and is currently used to treat a wider range of conditions than its original indication. The original Grammont-style RSA revolutionized shoulder arthroplasty but had several limitations, including scapular notching and reduced rotational motion. This review discusses the evolution of RSA design, particularly the development of a lateralized center of rotation constructs, which aims to improve all the disadvantages associated with the Grammont-style design and more closely reproduce the native anatomy in order to improve patient outcomes in an expanded context of pathologies.

## 1. Introduction

Since its introduction in the 1980s, reverse shoulder arthroplasty (RSA) has become an increasingly common surgical procedure of the shoulder, comprising 44.69% of shoulder arthroplasties since 2015 [1]. The Grammont Delta III implant is largely considered to be the first commercially successful RSA prosthesis and was a pivotal advancement in the surgical treatment of rotator cuff arthropathy (RCA). As innovation and modifications were made to the RSA prosthesis design, this allowed for the use of RSA for the management of a wider spectrum of disease beyond rotator cuff arthropathy. Current RSA indications include glenohumeral arthritis with eccentric glenoid wear, three- and four-part proximal humerus fracture, revision shoulder arthroplasty, and oncologic reconstruction [2]. Glenohumeral osteoarthritis with an intact rotator cuff (GHOA) represented 54% of the indications for RSA between 2016 and 2020 [3], introducing a new wave of pathology for which the ideal combination of surgical and implant factors is debated. Thus, the theory of an “anatomic RSA” aims to place the center of rotation (COR) more closely to its native position to optimize muscle length tension curves, lines of action, and the moment arms of all intact musculature around the shoulder. This is compared to previous RSA models where the COR is medialized and distalized to focus solely on deltoid optimization.

## 2. Grammont Design

**Figure 1:**
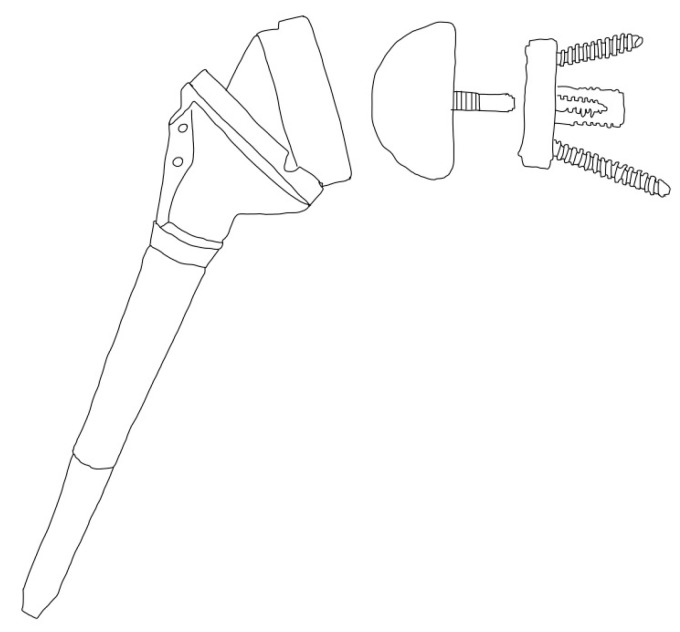
Delta III reverse shoulder arthroplasty prosthesis.

In the early 1970s, patients with severe glenohumeral osteoarthritis and concomitant rotator cuff deficiency were unable to obtain good functional outcomes and pain relief, with previous attempts at managing this problem including the use of hemiarthroplasty and anatomic total shoulder arthroplasty [4]. Dr. Neer attempted to solve this problem with a constrained prosthesis design that placed the COR in a lateralized position in comparison to the native humerus, but despite multiple attempts, this prosthesis design was abandoned due to high rates of glenoid implant failure [5,6,7]. Dr. Grammont had been studying the relationship between the balance of the supraspinatus–deltoid couple and the position of the prosthesis COR. He proposed that the optimization of the deltoid was imperative to obtaining good functional outcomes in this specific set of patients. With that in mind, the “Trompette” prosthesis was introduced in 1985. It was composed of a cemented glenoid component that consisted of two-thirds of a 44 mm diameter sphere that placed the COR in a medialized position in comparison to the native humerus and a cemented humeral polyethylene cone [8]. The initial outcomes with this prosthesis were successful, as reported in his initial series with a 6 month follow-up [9], but he later reported an unacceptable rate of glenoid component loosening. To address this concern, he further medialized the COR by changing the glenosphere component from two-thirds of a sphere to a hemisphere, placing it directly at the glenoid bone implant interface. Additionally, he substituted the cemented baseplate for a central press-fit peg baseplate with two 3.5 mm divergent screws [6,8,10]. These two modifications led to the Delta III prosthesis, which consists of a medialized COR at the glenoid-implant interface and a valgus, 155° humeral neck shaft angle (NSA) (Figure 1).

This non-anatomic RSA design was based on three hypotheses for the management of RCA. First, it established a fixed COR with congruent joint surfaces to stabilize the glenohumeral joint. The glenosphere acted as a fixed mechanical fulcrum, preventing proximal humeral migration and allowing for the conversion of the deltoid force to the abduction torque. This effectively compensated for the lost compressive mechanism in rotator cuff-deficient shoulders [11,12]. Second, the medialized COR increased the deltoid moment arm and decreased torque at the glenoid–-bone interface. This was achieved with the medial and inferior placement of the prosthesis, facilitating a reduced force necessary to initiate movement [13,14,15], while the decreased torque at the implant–bone interface decreased the risk of glenoid loosening. Third, the humerus was distalized relative to the glenoid to restore deltoid tension [6,10]. Despite the Grammont RSA achieving improvements in forward active elevation and decreasing pain [10,16], some important drawbacks remained.

There are some well-documented complications with the Grammont-style RSA prostheses. Scapular notching is a term used to describe an erosive lesion of the axillary border of the scapular neck that occurs when the medial rim of the humeral implant contacts the scapular during shoulder abduction. The reported incidence of this phenomenon varies widely, ranging from 4.6% to 96% [17,18], and has been associated with poor long-term functional outcomes [19] and increased number of complications, including aseptic glenoid loosening, persistent pain, and periprosthetic fracture [20]. The variable incidence of scapular notching is often multifactorial, including influences from preoperative diagnosis, glenoid wear/medialization, and implant positioning. A second-generation Delta prosthesis was introduced (Delta Xtend) to try to overcome this limitation, incorporating a smaller baseplate and an eccentric glenosphere, while maintaining the Grammont medialization concept [21,22]. Alberio et al. [23] have recently compared both first- and second-generation Delta prostheses, reporting a dramatic reduction in scapular notching from 100%, associated with the first generation, to 22%, associated with the second generation. These modifications improved prosthesis use for not only RCA, but also a wider range of indications now seen in practice [24].

Another common complication associated with Grammont-style RSA prostheses is the loss of rotational motion [25,26]. This disadvantage is, in part, a result of the medialization of the COR, which leads to an alteration in the length–tension relationship of the muscles responsible for rotational movement [21,27]. Several surgical and prosthetic factors have been established to influence the degree of rotational motion restoration that can be achieved, including the humeral NSA as well as the glenosphere diameter and lateralization, among others. While Grammont RSA revolutionized the treatment of RSA, both notching and rotational motion provided areas for improvement and innovation, particularly in rotator cuff-intact patients.

## 3. Introduction of a Lateralized RSA

To expand upon foundational Grammont-style RSA constructs, a novel RSA prosthesis was designed with a more lateralized COR, striving to more closely reproduce the native humerus COR. Frankle et al. [28] reported the results of using this alternative design on sixty RCA patients. The patients had similar improvements in active elevation and improved rotational motion in comparison to the Delta III prosthesis, without any cases of scapular notching. However, 12% of patients experienced early glenoid baseplate failure, later attributed to the lateralized COR increasing stresses at the baseplate bone–interface [29]. These failures rapidly decreased after two modifications. First, baseplate peripheral screws were increased from 3.5 mm to 5.0 mm locking screws, improving initial fixation and minimizing micromotion [29]. Second, the baseplates were placed in neutral or inferior tilt, as opposed to superior tilt, to improve compression across the bone–implant interface [30]. By modifying baseplate positioning, with the use of a central compressive screw with 5.0 mm peripheral locking screws, a reduction in premature mechanical failure in lateralized COR RSA constructs was seen, with no cases being reported at the two-year follow-up [31] and a 91% survivorship at the ten-year follow-up [32]. Although many studies have raised concerns that by using a lateralized COR there was an increased rate of aseptic glenoid loosening due to the increased torque and shear forces [28,29,33,34], a recent systematic review published by Rojas et al. [35] involving 103 studies and 6583 RSAs reported no significant differences between medialized and lateralized glenoid designs.

Lateralized COR RSA constructs have increased in popularity and can be achieved through different combinations and modifications of the prosthesis components.

### 3.1. Glenoid-Sided Lateralization

**Figure 2:**
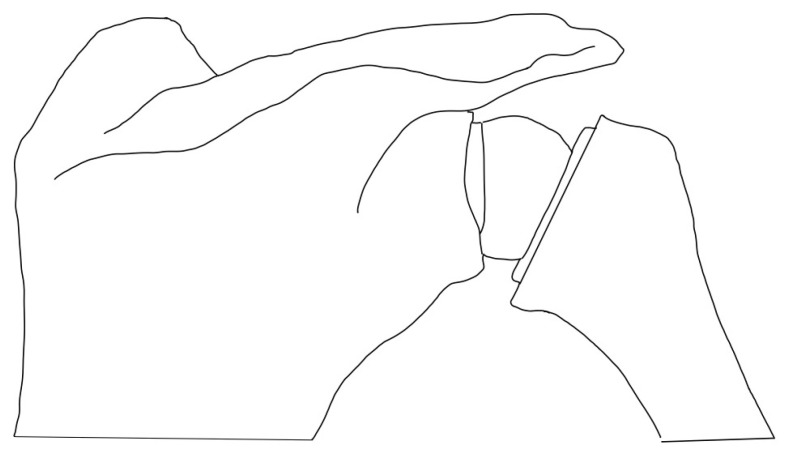
Glenoid-sided lateralization.

RSA constructs with COR > 5 mm from the glenoid face through modifications to the glenoid component are deemed lateralized on the glenoid side [36] (Figure 2). Several options are available to achieve this. Increasing glenosphere thickness changes the major spherical radius, leading to a lateralized placement of the COR. Berhouet et al. [37] performed a cadaveric study where they reported greater rotational range of motion (ROM) when using a larger glenosphere (42 mm) in comparison to a smaller glenosphere (36 mm). Similar results were shown by Mollon et al. [38] when they compared the impact of glenosphere size on patient clinical outcomes, reporting greater improvements in active forward elevation and external rotation when using the larger glenosphere. More recently, King et al. [39] showed that there were no differences in postoperative ROM and outcome scores between using a lateralized glenosphere and a standard glenosphere. Currently, no single study has been able to demonstrate the ideal glenosphere size to optimize patient performance.

Baseplate modifications provide an alternative option for glenoid-sided lateralization through the use of bony metal augmentation. Bony-Increased offset Reverse Shoulder Arthroplasty (BIO-RSA) was introduced by Boileau et al. [25] in the context of posterior glenoid deficiencies. Initial outcomes with this technique reported a 98% healing rate of the autograph with no component loosening at 28 months and a 19% rate of scapular notching, with long-term outcomes yielding similar results [25,40]. Theoretically, with the use of a bone graph, the COR would be maintained at the glenoid bone–implant interface while still lateralizing the arthroplasty construct, decreasing shear forces and reducing the risk of glenoid loosening associated with early lateralized constructs [35,41]. Additionally, BIO-RSA may also present some additional challenges for patients with medialized wear patterns in which large grafts may be required to restore the joint line. The threshold for lateralization with BIO-RSA is about 5 mm, whereas glenosphere lateralization can achieve 10-15 mm of lateralization [29,42,43]. BIO-RSA has also been associated with higher rates of scapular stress fractures, with patients suffering from decreased functional outcomes [44]. Lastly, some authors have raised concerns about the increased risk of baseplate loosening associated with the use of bone graft. A recent comparative study between BIO-RSA and metal-augmented baseplates found that 36.4% of BIO-RSA patients showed radiolucent lines around the bone graft, and 34.1% experienced decreased graft thickness [45].

More recently, metallic augmented baseplates have been introduced for the management of glenoid bone deficiency in RSA. This option eliminates the risk of graft failure to incorporate graft resorption. A systematic review published by Ghanta et al. [46] showed that with the use of a metallic-augmented baseplate, surgeons were able to provide positive clinical and functional outcomes at a short-term follow-up. However, findings on long-term performance are still lacking currently in the literature.

Several authors have looked into the influence of glenoid lateralization in the restoration of active internal rotation after RSA. Li et al. [26] reported their results on a computer model that evaluated how different glenoid-sided implant positions would influence impingement-free external and internal rotation after RSA. They reported that glenosphere positioning did have a significant effect, with optimal positioning being described as inferiorly translated, inferiorly tilted, and with 10 mm of lateralization. Subsequent studies have further explored this relationship, with similar results being reported [47,48]. More recently, Werner and colleagues [49] performed a study involving 455 patients where they compared the grade of active internal rotation based on the amount of glenoid-implant lateralization. They found that the greatest improvement in active internal rotation was seen with 6 to 8 mm of glenoid-sided lateralization.

### 3.2. Humeral-Sided Lateralization

**Figure 3:**
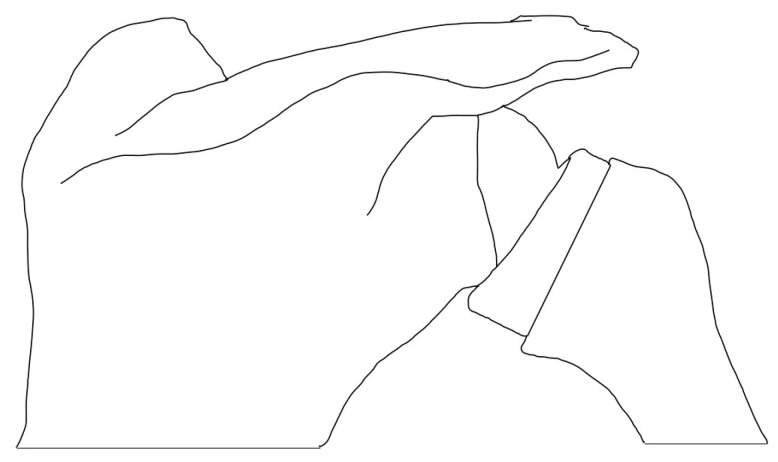
Humeral-sided lateralization.

The original polyethylene humeral inlay from the Grammont reverse prosthesis was designed with a 155° non-anatomic NSA, with modern designs exploring alternative NSAs. NSA has been associated with having the largest effect on reducing inferior scapular impingement [50], achieving this through more vertical inclinations (Figure 3). With the use of three-dimensional templating, Werner et al. [51] analyzed the influence of NSA and bony COR on ROM. They concluded that a more valgus NSA increased impingement-free ROM, with the best results being achieved with a configuration of a 135° NSA and 5 mm of glenoid lateralization. A systematic review of 2,222 shoulders found a 16.8% incidence of scapular notching with a 155° inclination prosthesis compared to a 2.8% incidence with a 135° inclination prosthesis [52].

Onlay humeral component designs lateralized the humerus, since the humeral tray rests on top of the humeral stem. They have been associated with increased tension in the rotator cuff and the lengthening of the deltoid muscle [2,14]. Jackson et al. [53] performed a systematic review comparing outcomes and complications when using an onlay or an inlay humeral component for the RSA. They showed that both options were associated with improvements in the ROM, with onlay humeral components showing greater external rotation and a lower rate of scapular notching. Increased lateral offset can also be achieved on the humeral side with the use of a curved stem, as demonstrated by Läderman et al. [54]; however, unlike other options, lateralization through a curved stem is predetermined by the implant design, limiting intraoperative modifications.

## 4. Defining an Anatomic Reverse Shoulder Arthroplasty

The use of the term “Anatomic Reverse Shoulder Arthroplasty” might sound somewhat counterintuitive, since the defining characteristic of an RSA prosthesis is the non-anatomic reversed ball-and-socket construct. However, the benefit of achieving a more anatomical RSA is that by obtaining a reconstruction that is able to closely resemble the native anatomy relationship, it would allow the muscles to remain in their designed functional position and purpose while simultaneously maintaining a more constrained reverse articulation for maximum stability. Recent studies have challenged the notion that RSA consistently increases deltoid efficiency through moment arm enhancement, with the current literature on the impact on overall deltoid efficiency remaining unclear [55,56,57,58], suggesting that factors beyond moment arm play a crucial role in shoulder biomechanics post-RSA. By restoring the pre-morbid humero-scapular relationship, and by matching the COR of the native humerus to the RSA pivot point, an improved construct can be achieved that could potentially achieve optimal outcomes.

This principle is most clearly illustrated in setting the RSA for GHOA with the intact rotator cuff, where RSA has shown similar short-term patient-reported outcomes in anatomic total shoulder [59,60]. Additionally, RSA has shown lower complication and reoperation rates with higher patient satisfaction, with some long-term studies suggesting that RSA may outperform TSA in terms of durability and patient satisfaction [61]. Moreover, this principle can be applied to any RSA indication to improve the ability of the shoulder musculature to act in a similar manner to their designed function.

### 4.1. Maximizing Impingement-Free ROM

One disadvantageous biomechanical sequelae of a reverse articulation is the impingement of the proximal humerus and/or humeral socket on various regions of the scapula. To create a shoulder with the largest potential for motion, maximizing impingement-free ROM of the RSA is critical, and several factors need to be taken into consideration. Gutierrez et al. [62] used a computer model to demonstrate that glenoid component lateralization has the greatest impact on impingement-free abduction ROM, while the avoidance of an adduction deficit was achieved by the use of a more varus (130°) NSA. This is supported by the Keener et al. [48] study, where a computer software analysis was performed on 10 shoulder computed tomography images, reporting that maximum ROM is achieved with 10 mm baseplate lateralization in combination with a varus (135°) NSA. Furthermore, Werner et al. [51] reported that decreasing humeral NSA demonstrated a significant increase in impingement-free ROM, where a 135° NSA with +5 mm of glenoid lateralization provided the best overall ROM. Therefore, the use of glenoid lateralization (+5 to +10 mm) and a more anatomic NSA (135°) in comparison to the Grammont-style prosthesis (+0 mm and 155°) has consistently been shown to maximize the impingement-free ROM of the RSA construct.

### 4.2. Optimizing Shoulder Muscle Length–Tension Relationship

While impingement-free ROM is required for passive motion at the glenohumeral joint, the active and functional motion of the joint requires adequate muscle force in the desired force vector to power the shoulder. Furthermore, achieving ideal soft-tissue tension in RSA has been widely recognized as being critical in optimizing functional outcomes and minimizing complications [63]. Inability to achieve an adequate deltoid and rotator cuff can potentially lead to instability, reduced ROM, and weakness [64], while excessive tension has been associated with increased pain and polyethylene wear [65,66]. Much of the literature has focused on the moment arm as the most important feature facilitating muscle strength and therefore motion [56,67,68]; however, there is little information on how the RSA alters muscle geometry and thus muscle function. RSA implants have been developed under the hypothesis that optimal shoulder function would occur by maximizing the deltoid moment arm [56,63,67]. However, there is evidence of there being unintended biomechanical consequences of maximizing the deltoid moment arm, including reductions in the supraspinatus moment arm as well as substantial alterations in the muscle fiber lengths of the deltoid and the rotator cuff musculature [69]. These consequences represent a tradeoff in maximizing the deltoid moment arm, which may compromise both rotator cuff and deltoid muscle performance, and therefore shoulder function. This has been highlighted by Levin et al. [70], where a geometric biomechanical model was used to evaluate how three different implant designs affected the moment arm and muscle fiber lengths of the deltoid and supraspinatus through functional scapular plane abduction, including the following: (1) a lateralized glenosphere with an inlay 135° humeral component (LGMH), (2) a medialized glenosphere with an onlay 145° humeral component (MGLH), and (3) a medialized glenosphere with an inlay 155° humerus (MGMH). The results demonstrated that the MGLH and MGMH had substantial deltoid muscle fiber overlengthening, shifting the operating range of this muscle outside of the optimal portion of its force–length curve (Blick’s Curve), whereas the LGMH design maintained a deltoid muscle fiber length similar to the native force–length curve. Additionally, MGLH decreased the supraspinatus moment arm by 59% compared to just a 14% decrease in the LGMH design. Therefore, lateralizing the glenoid and using an inlay 135° humeral component provides a geometric reconstruction that best replicates the restoration of muscle fiber lengths, such that it remains within the optimal force–length curve and could be achieved with a more modest increased in deltoid moment arm [70].

More recently, this computational model has been expanded to evaluate all four rotator cuff muscles along with the anterior, middle, and posterior heads of the deltoid, and to analyze which combination of surgical and implant-design-related parameters most closely restored the anatomic muscle–tendon lengths in three different shoulder sizes [71]. Surgical parameters, including glenosphere position (centered vs. inferior) and humeral offset relative to the anatomic neck plane (+0 mm vs. +5 mm vs. +10 mm), were combined with different implant designs, including glenosphere size (30 mm vs. 36 mm vs. 42 mm), glenosphere lateralization (+0 mm vs. +5 mm vs. +10 mm), and NSA (135° vs. 145° vs. 155°), leading to 486 RSA–shoulder size combinations. The results demonstrated that the configurations that most closely restored the anatomic muscle–tendon lengths were as follows: (1) 30 mm glenosphere in a centered position with 5 mm of glenoid lateralization associated with +0 mm of humeral offset and 135° NSA for a small size shoulder; (2) 36 mm glenosphere in a centered position with 5 mm of glenoid lateralization associated with +0 mm of humeral offset and 135° NSA for a medium size shoulder; and (3) 30 mm glenosphere in a centered position with 10 mm of glenoid lateralization associated with +0 mm of humeral offset and 135° NSA for larger shoulders. The most important surgical parameter associated with the restoration of native muscle–tendon length was humeral offset, favoring a humeral socket placed at the anatomic neck plane. Additionally, the most important implant design parameter associated with the restoration of the native muscle–tendon length was the humeral NSA, favoring a 135° NSA. Overall, smaller glenospheres with a lateralized glenosphere, a humeral socket placed at the anatomic neck plane, and an anatomic 135° NSA best restores native deltoid and rotator cuff muscle–tendon lengths in RSA. The “anatomic RTSA” is thus most closely achieved by using smaller glenospheres placed centrally on the glenoid with a lateralized glenosphere, a humeral socket placed at the anatomic neck plane, and an anatomic 135° NSA, as this combination of features best restores native deltoid and rotator cuff muscle–tendon lengths in RSA [71].

### 4.3. Long-Term Clinical Outcomes of a More Anatomical RSA

In addition to the biomechanical benefits of improved impingement-free ROM, as well as the restoration of the anatomic muscle–tendon lengths of the rotator cuff and deltoid, the lateralized glenoid with an inlay 135° NSA has excellent and durable long-term clinical outcomes at the minimum 10-year follow-up, with patients maintaining their postoperative gains in ASES scores, SST scores, and improvements in ROM [32]. This contrasts with Sirveaux et al. [27] and Guery et al. [72], in which a Grammont-style prosthesis was used, which demonstrated long-term functional deterioration and increase in pain levels after the 6-year follow-up time point. In theory, by providing a more anatomical RSA reconstruction, the detrimental effect of overlengthening on muscle function can be mitigated and more durable clinical outcomes can possibly be achieved.

## 5. Future Direction of Patient-Specific Reverse Shoulder Arthroplasty

Since its introduction, RSA has become the most common shoulder arthroplasty performed, with broad indications spanning degenerative, traumatic, and oncologic pathologies [2]. Despite significant improvements in scapular notching, rotational motion, and clinical outcomes [28,29,31,32,54], there remains heterogeneity in individual patient outcomes. A more anatomical RSA has evolved from the first commercially successful RSA prosthesis, the Grammont-style prosthesis, to one that more closely replicates the native shoulder from a biomechanical perspective, while providing a stable fulcrum to achieve motion through the shoulder. Impingement-free ROM and biomechanical studies assessing muscle force–length relationships support the benefits of a more anatomical design, consisting of a lateralized glenosphere and an inlay 135° humeral component [48,50,51]. With the advancements in complex kinematic biomechanical simulation techniques and applications of artificial intelligence in enhanced automated patient-specific preoperative assessment of bone and muscle pathology, progress will be made towards an optimal RSA construct and surgical placement for any given patient and pathology. Furthermore, a more precise understanding of the optimal implant design and positioning will potentially allow for benefits in navigation and robotic surgery to improve patient outcomes, as these technologies will undoubtedly enhance the precision of component placement.

## References

[1] American Academy of Orthopaedic Surgeons (2023). Shoulder and Elbow Registry Annual Report.

[2] Kozak T., Bauer S., Walch G., Al-Karawi S., Blakeney W. (2021). An update on reverse total shoulder arthroplasty: Current indications, new designs, same old problems. EFORT Open Rev..

[3] Mayfield C.K., Korber S.S., Hwang N.M., Bolia I.K., Gamradt S.C., Weber A.E., Liu J.N., Petrigliano F.A. (2023). Volume, indications, and number of surgeons performing reverse total shoulder arthroplasty continue to expand: A nationwide cohort analysis from 2016–2020. JSES Int..

[4] Grammont P.M., Baulot E. (1993). Delta shoulder prosthesis for rotator cuff rupture. Orthopedics.

[5] Neer N.C. (1990). Shoulder Reconstruction.

[6] Flatow E.L., Harrison A.K. (2011). A history of reverse total shoulder arthroplasty. Clin. Orthop. Relat. Res..

[7] Thon S.G., Seidl A.J., Bravman J.T., McCarty E.C., Savoie F.H., Frank R.M. (2020). Advances and Update on Reverse Total Shoulder Arthroplasty. Curr. Rev. Musculoskelet. Med..

[8] Baulot E., Sirveaux F., Boileau P. (2011). Grammont’s idea: The story of Paul Grammont’s functional surgery concept and the development of the reverse principle. Clin. Orthop. Relat. Res..

[9] Grammont P., Trouilloud P., Laffay J., Deries X. (1987). Study and development of a new shoulder prosthesis. Rhumatologie.

[10] Boileau P., Watkinson D.J., Hatzidakis A.M., Balg F. (2005). Grammont reverse prosthesis: Design, rationale, and biomechanics. J. Shoulder Elb. Surg..

[11] Roche C.P. (2022). Reverse Shoulder Arthroplasty Biomechanics. J. Funct. Morphol. Kinesiol..

[12] Rugg C.M., Coughlan M.J., Lansdown D.A. (2019). Reverse Total Shoulder Arthroplasty: Biomechanics and Indications. Curr. Rev. Musculoskelet. Med..

[13] Henninger H.B., Barg A., Anderson A.E., Bachus K.N., Burks R.T., Tashjian R.Z. (2012). Effect of lateral offset center of rotation in reverse total shoulder arthroplasty: A biomechanical study. J. Shoulder Elb. Surg..

[14] Roche C.P., Diep P., Hamilton M., Crosby L.A., Flurin P.H., Wright T.W., Zuckerman J.D., Routman H.D. (2013). Impact of inferior glenoid tilt, humeral retroversion, bone grafting, and design parameters on muscle length and deltoid wrapping in reverse shoulder arthroplasty. Bull. Hosp. Jt. Dis..

[15] Otis J.C., Jiang C.C., Wickiewicz T.L., Peterson M.G., Warren R.F., Santner T.J. (1994). Changes in the moment arms of the rotator cuff and deltoid muscles with abduction and rotation. J. Bone Jt. Surg. Am..

[16] Boileau P., Watkinson D., Hatzidakis A.M., Hovorka I. (2006). Neer Award 2005: The Grammont reverse shoulder prosthesis: Results in cuff tear arthritis, fracture sequelae, and revision arthroplasty. J. Shoulder Elb. Surg..

[17] Friedman R.J., Barcel D.A., Eichinger J.K. (2019). Scapular Notching in Reverse Total Shoulder Arthroplasty. J. Am. Acad. Orthop. Surg..

[18] Alentorn-Geli E., Samitier G., Torrens C., Wright T.W. (2015). Reverse shoulder arthroplasty. Part 2: Systematic review of reoperations, revisions, problems, and complications. Int. J. Shoulder Surg..

[19] Jang Y.H., Lee J.H., Kim S.H. (2020). Effect of scapular notching on clinical outcomes after reverse total shoulder arthroplasty. Bone Jt. J..

[20] Mollon B., Mahure S.A., Roche C.P., Zuckerman J.D. (2017). Impact of scapular notching on clinical outcomes after reverse total shoulder arthroplasty: An analysis of 476 shoulders. J. Shoulder Elb. Surg..

[21] Nyffeler R.W., Werner C.M., Gerber C. (2005). Biomechanical relevance of glenoid component positioning in the reverse Delta III total shoulder prosthesis. J. Shoulder Elb. Surg..

[22] de Wilde L.F., Poncet D., Middernacht B., Ekelund A. (2010). Prosthetic overhang is the most effective way to prevent scapular conflict in a reverse total shoulder prosthesis. Acta Orthop..

[23] Alberio R.L., Landrino M., Fornara P., Grassi F.A. (2019). Short-Term Outcomes of the Grammont Reverse Shoulder Arthroplasty: Comparison between First and Second Generation Delta Prosthesis. Joints.

[24] Gruber M.D., Kirloskar K.M., Werner B.C., Ladermann A., Denard P.J. (2022). Factors Associated with Internal Rotation After Reverse Shoulder Arthroplasty: A Narrative Review. JSES Rev. Rep. Tech..

[25] Boileau P., Moineau G., Roussanne Y., O’Shea K. (2011). Bony increased-offset reversed shoulder arthroplasty: Minimizing scapular impingement while maximizing glenoid fixation. Clin. Orthop. Relat. Res..

[26] Li X., Knutson Z., Choi D., Lobatto D., Lipman J., Craig E.V., Warren R.F., Gulotta L.V. (2013). Effects of glenosphere positioning on impingement-free internal and external rotation after reverse total shoulder arthroplasty. J. Shoulder Elb. Surg..

[27] Sirveaux F., Favard L., Oudet D., Huquet D., Walch G., Mole D. (2004). Grammont inverted total shoulder arthroplasty in the treatment of glenohumeral osteoarthritis with massive rupture of the cuff. Results of a multicentre study of 80 shoulders. J. Bone Jt. Surg. Br..

[28] Frankle M., Siegal S., Pupello D., Saleem A., Mighell M., Vasey M. (2005). The Reverse Shoulder Prosthesis for glenohumeral arthritis associated with severe rotator cuff deficiency. A minimum two-year follow-up study of sixty patients. J. Bone Jt. Surg. Am..

[29] Harman M., Frankle M., Vasey M., Banks S. (2005). Initial glenoid component fixation in "reverse" total shoulder arthroplasty: A biomechanical evaluation. J. Shoulder Elb. Surg..

[30] Gutierrez S., Greiwe R.M., Frankle M.A., Siegal S., Lee W.E. (2007). Biomechanical comparison of component position and hardware failure in the reverse shoulder prosthesis. J. Shoulder Elb. Surg..

[31] Cuff D., Pupello D., Virani N., Levy J., Frankle M. (2008). Reverse Shoulder Arthroplasty for the Treatment of Rotator Cuff Deficiency. JBJS.

[32] Cuff D.J., Pupello D.R., Santoni B.G., Clark R.E., Frankle M.A. (2017). Reverse Shoulder Arthroplasty for the Treatment of Rotator Cuff Deficiency: A Concise Follow-up, at a Minimum of 10 Years, of Previous Reports. JBJS.

[33] Lawrence C., Williams G.R., Namdari S. (2016). Influence of Glenosphere Design on Outcomes and Complications of Reverse Arthroplasty: A Systematic Review. Clin. Orthop. Surg..

[34] Zumstein M.A., Pinedo M., Old J., Boileau P. (2011). Problems, complications, reoperations, and revisions in reverse total shoulder arthroplasty: A systematic review. J. Shoulder Elb. Surg..

[35] Rojas J., Choi K., Joseph J., Srikumaran U., McFarland E.G. (2019). Aseptic Glenoid Baseplate Loosening After Reverse Total Shoulder Arthroplasty: A Systematic Review and Meta-Analysis. JBJS Rev..

[36] Routman H.D., Flurin P.H., Wright T.W., Zuckerman J.D., Hamilton M.A., Roche C.P. (2015). Reverse Shoulder Arthroplasty Prosthesis Design Classification System. Bull. Hosp. Jt. Dis..

[37] Berhouet J., Garaud P., Favard L. (2013). Influence of glenoid component design and humeral component retroversion on internal and external rotation in reverse shoulder arthroplasty: A cadaver study. Orthop. Traumatol. Surg. Res..

[38] Mollon B., Mahure S.A., Roche C.P., Zuckerman J.D. (2016). Impact of glenosphere size on clinical outcomes after reverse total shoulder arthroplasty: An analysis of 297 shoulders. J. Shoulder Elb. Surg..

[39] King J.J., Hones K.M., Wright T.W., Roche C., Zuckerman J.D., Flurin P.H., Schoch B.S. (2023). Does isolated glenosphere lateralization affect outcomes in reverse shoulder arthroplasty?. Orthop. Traumatol. Surg. Res..

[40] Boileau P., Morin-Salvo N., Bessiere C., Chelli M., Gauci M.O., Lemmex D.B. (2020). Bony increased-offset-reverse shoulder arthroplasty: 5 to 10 years’ follow-up. J. Shoulder Elb. Surg..

[41] Dimock R., Fathi Elabd M., Imam M., Middleton M., Godeneche A., Narvani A.A. (2021). Bony increased-offset reverse shoulder arthroplasty: A meta-analysis of the available evidence. Shoulder Elb..

[42] Jasty M., Bragdon C., Burke D., O’Connor D., Lowenstein J., Harris W.H. (1997). In vivo skeletal responses to porous-surfaced implants subjected to small induced motions. J. Bone Jt. Surg. Am..

[43] Denard P.J., Lederman E., Parsons B.O., Romeo A.A. (2017). Finite element analysis of glenoid-sided lateralization in reverse shoulder arthroplasty. J. Orthop. Res..

[44] Kirzner N., Paul E., Moaveni A. (2018). Reverse shoulder arthroplasty vs BIO-RSA: Clinical and radiographic outcomes at short term follow-up. J. Orthop. Surg. Res..

[45] Merolla G., Giorgini A., Bonfatti R., Micheloni G.M., Negri A., Catani F., Tarallo L., Paladini P., Porcellini G. (2023). BIO-RSA vs. metal-augmented baseplate in shoulder osteoarthritis with multiplanar glenoid deformity: A comparative study of radiographic findings and patient outcomes. J. Shoulder Elb. Surg..

[46] Ghanta R.B., Tsay E.L., Feeley B. (2023). Augmented baseplates in reverse shoulder arthroplasty: A systematic review of outcomes and complications. JSES Rev. Rep. Tech..

[47] Kramer M., Baunker A., Wellmann M., Hurschler C., Smith T. (2016). Implant impingement during internal rotation after reverse shoulder arthroplasty. The effect of implant configuration and scapula anatomy: A biomechanical study. Clin. Biomech..

[48] Keener J.D., Patterson B.M., Orvets N., Aleem A.W., Chamberlain A.M. (2018). Optimizing reverse shoulder arthroplasty component position in the setting of advanced arthritis with posterior glenoid erosion: A computer-enhanced range of motion analysis. J. Shoulder Elb. Surg..

[49] Werner B.C., Lederman E., Gobezie R., Denard P.J. (2021). Glenoid lateralization influences active internal rotation after reverse shoulder arthroplasty. J. Shoulder Elb. Surg..

[50] Gutierrez S., Levy J.C., Frankle M.A., Cuff D., Keller T.S., Pupello D.R., Lee W.E. (2008). Evaluation of abduction range of motion and avoidance of inferior scapular impingement in a reverse shoulder model. J. Shoulder Elb. Surg..

[51] Werner B.S., Chaoui J., Walch G. (2017). The influence of humeral neck shaft angle and glenoid lateralization on range of motion in reverse shoulder arthroplasty. J. Shoulder Elb. Surg..

[52] Erickson B.J., Frank R.M., Harris J.D., Mall N., Romeo A.A. (2015). The influence of humeral head inclination in reverse total shoulder arthroplasty: A systematic review. J. Shoulder Elb. Surg..

[53] Jackson G.R., Meade J., Young B.L., Trofa D.P., Schiffern S.C., Hamid N., Saltzman B.M. (2023). Onlay versus inlay humeral components in reverse shoulder arthroplasty: A systematic review and meta-analysis. Shoulder Elb..

[54] Ladermann A., Denard P.J., Boileau P., Farron A., Deransart P., Terrier A., Ston J., Walch G. (2015). Effect of humeral stem design on humeral position and range of motion in reverse shoulder arthroplasty. Int. Orthop..

[55] Roche C.P., Hamilton M.A., Diep P., Wright T.W., Flurin P.H., Zuckerman J.D., Routman H.D. (2015). Optimizing Deltoid Efficiency with Reverse Shoulder Arthroplasty Using a Novel Inset Center of Rotation Glenosphere Design. Bull. Hosp. Jt. Dis..

[56] Ackland D.C., Roshan-Zamir S., Richardson M., Pandy M.G. (2010). Moment Arms of the Shoulder Musculature After Reverse Total Shoulder Arthroplasty. JBJS.

[57] Martinez L., Machefert M., Poirier T., Matsoukis J., Billuart F. (2021). Analysis of the coaptation role of the deltoid in reverse shoulder arthroplasty. A preliminary biomechanical study. PLoS ONE.

[58] Scalise J., Jaczynski A., Jacofsky M. (2016). The effect of glenosphere diameter and eccentricity on deltoid power in reverse shoulder arthroplasty. Bone Jt. J..

[59] Kirsch J.M., Puzzitiello R.N., Swanson D., Le K., Hart P.A., Churchill R., Elhassan B., Warner J.J.P., Jawa A. (2022). Outcomes After Anatomic and Reverse Shoulder Arthroplasty for the Treatment of Glenohumeral Osteoarthritis: A Propensity Score-Matched Analysis. J. Bone Jt. Surg. Am..

[60] Flurin P.H., Marczuk Y., Janout M., Wright T.W., Zuckerman J., Roche C.P. (2013). Comparison of outcomes using anatomic and reverse total shoulder arthroplasty. Bull Hosp. Jt. Dis..

[61] Schoch B.S., King J.J., Zuckerman J., Wright T.W., Roche C., Flurin P.H. (2021). Anatomic versus reverse shoulder arthroplasty: A mid-term follow-up comparison. Shoulder Elb..

[62] Gutierrez S., Comiskey C.A.t., Luo Z.P., Pupello D.R., Frankle M.A. (2008). Range of impingement-free abduction and adduction deficit after reverse shoulder arthroplasty. Hierarchy of surgical and implant-design-related factors. J. Bone Jt. Surg. Am..

[63] Bauer S., Blakeney W.G., Wang A.W., Ernstbrunner L., Corbaz J., Werthel J.D. (2023). Challenges for Optimization of Reverse Shoulder Arthroplasty Part II: Subacromial Space, Scapular Posture, Moment Arms and Muscle Tensioning. J. Clin. Med..

[64] Ladermann A., Edwards T.B., Walch G. (2014). Arm lengthening after reverse shoulder arthroplasty: A review. Int. Orthop..

[65] Giles J.W., Langohr G.D., Johnson J.A., Athwal G.S. (2015). Implant Design Variations in Reverse Total Shoulder Arthroplasty Influence the Required Deltoid Force and Resultant Joint Load. Clin. Orthop. Relat. Res..

[66] Langohr G.D., Giles J.W., Athwal G.S., Johnson J.A. (2015). The effect of glenosphere diameter in reverse shoulder arthroplasty on muscle force, joint load, and range of motion. J. Shoulder Elb. Surg..

[67] Hamilton M.A., Roche C.P., Diep P., Flurin P.H., Routman H.D. (2013). Effect of prosthesis design on muscle length and moment arms in reverse total shoulder arthroplasty. Bull. Hosp. Jt. Dis..

[68] Hamilton M.A., Diep P., Roche C., Flurin P.H., Wright T.W., Zuckerman J.D., Routman H. (2015). Effect of reverse shoulder design philosophy on muscle moment arms. J. Orthop. Res..

[69] Sabesan V.J., Lombardo D., Josserand D., Buzas D., Jelsema T., Petersen-Fitts G.R., Wiater J.M. (2016). The effect of deltoid lengthening on functional outcome for reverse shoulder arthroplasty. Musculoskelet. Surg..

[70] Levin J.M., Pugliese M., Gobbi F., Pandy M.G., Di Giacomo G., Frankle M.A. (2023). Impact of reverse shoulder arthroplasty design and patient shoulder size on moment arms and muscle fiber lengths in shoulder abductors. J. Shoulder Elb. Surg..

[71] Levin J.M., Gobbi F., Pandy M.G., Di Giacomo G., Frankle M.A. (2024). Optimizing Muscle-Tendon Lengths in Reverse Total Shoulder Arthroplasty: Evaluation of Surgical and Implant-Design-Related Parameters. J. Bone Jt. Surg. Am..

[72] Guery J., Favard L., Sirveaux F., Oudet D., Mole D., Walch G. (2006). Reverse total shoulder arthroplasty. Survivorship analysis of eighty replacements followed for five to ten years. J. Bone Jt. Surg. Am..

